# Chromium-Modified Heterogeneous Bipolar Membrane: Structure, Characteristics, and Practical Application in Electrodialysis

**DOI:** 10.3390/membranes13020172

**Published:** 2023-01-31

**Authors:** Olga Kozaderova

**Affiliations:** 1 Faculty of Chemistry, Voronezh State University, 394018 Voronezh, Russia; kozaderova-olga@mail.ru; 2 Faculty of Ecology and Chemical Technology, Voronezh State University of Engineering Technologies, 394036 Voronezh, Russia

**Keywords:** heterogeneous bipolar membrane, ion-exchange membrane, chromium (III) hydroxide, modification

## Abstract

The modification of an MB-2 bipolar ion exchange membrane with chromium (III) hydroxide was carried out by a chemical method, namely, by the sequential treatment of the membrane with a solution of chromium (III) salt and alkali. Data on the morphology, phase, and chemical composition of the modified membrane were obtained using scanning electron microscopy and energy-dispersive analysis. In particular, it was shown that the modifier was distributed in a layer 30–50 microns thick at the boundary of the cation- and anion-exchange layers of the bipolar membrane. The electrochemical behavior of the modified membrane in the process of sodium sulfate conversion was studied by measurements of the following characteristics: the current efficiency of the acid and base, the energy consumption of the process, and the degree of contamination of the target products with salt ions. It was shown that the resulting membrane has an alkali and acid yield of 61% and 57%, respectively. This is higher than the same yields for the industrial unmodified MB-2 membrane (38% and 30%). The results of this study demonstrated that the modified samples allowed obtaining a higher yield of acid and base, reducing the content of salt ions in the target products and also reducing the electricity consumption for obtaining a unit of the target product. The concentration dependences of the electrical conductivity of the MK-40 heterogeneous ion-exchange membrane, which is a cation-exchange layer of MB-2, in sodium sulfate solutions before and after its modification with chromium (III) oxide were obtained. A decrease in the specific electrical conductivity of the membrane with the introduction of a modifier was established. A quantitative assessment of the influence of the modifier on the current flow, volume fraction, and spatial orientation of the conductive phases of MK-40 was carried out using an extended three-wire model for the description of the model parameters of ion-exchange materials. When a modifying additive was introduced into MK-40, the fraction of the current passing through the inner solution and the intergel phase decreased. This was due to the substitution of part of the free solution in the pore volume by the modifier. A variant of the practical application of electrodialysis with the chromium-modified bipolar ion-exchange membranes is recommended.

## 1. Introduction

**Figure 1:**
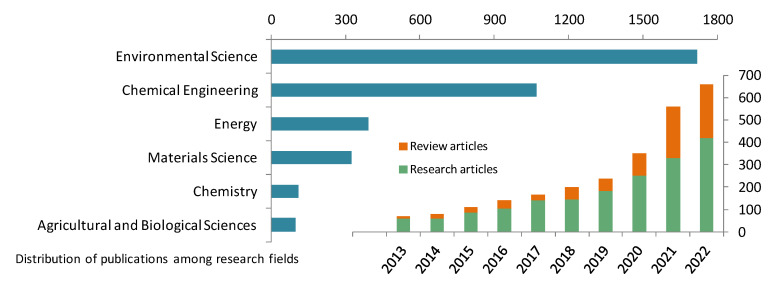
Research and review articles on the topic “Chromium-containing wastewater”. Data source: https://www.sciencedirect.com/ (accessed on 30 November 2022).

Over the past 10 years, research interest on the issue of chromium-containing wastewater treatment increased, which was due to the active use of chromium compounds in industrial processes and the improvement of existing methods as well as the appearance of new ones for the removal of the toxic metal (Figure 1). 

Industrial wastewater contains both trivalent Cr(III) ions and hexavalent Cr(VI) ions. Cr(VI) is considered more toxic than Cr(III) [1]. The maximum permissible concentration of chromium in drinking water recommended by the World Health Organization (WHO) is 0.05 ppm. Chromium-containing wastewater is treated using chemical precipitation and coagulation [2,3], electrocoagulation [4,5], electroreduction and membrane separation [6], ion exchange [7], and sorption methods [8,9,10,11,12].

The most common treatment of chromium-containing wastewater in the electroplating industry is a method based on the reduction of chromium (VI) followed by the precipitation of trivalent chromium [13,14]. This method is based on the use of relatively cheap reducing agents resulting in products of a lower toxicity class. Iron (II) salts for example, sulfates, are often used for the reduction of Cr_2_O_7_^2−^ to Cr^3+^:

K_2_Cr_2_O_7_ + 6FeSO_4_ + 7H_2_SO_4_ → Cr_2_(SO_4_)_3_ + 3Fe_2_(SO_4_)_2_ + K_2_SO_4_ + 7H_2_O. (1)

The reduction is carried out in an acidic medium at pH = 2–2.5. This requires the additional acidification of wastewater with a 10–15% sulfuric acid solution. For acidic solutions, instead of iron (II) salts, sodium sulfite or bisulfite is used:

K_2_Cr_2_O_7_ + 3Na_2_SO_3_ + 4H_2_SO_4_ = Cr_2_(SO_4_)_3_ + K_2_SO_4_ + 3Na_2_SO_4_ +4H_2_O. (2)

After the reduction of chromium, wastewater is neutralized with limewater to a pH of 8.5–9. Hydroxides are released as the result of neutralization:

Cr_2_(SO_4_)_3_ + 3Ca(OH)_2_ = 3CaSO_4_↓ + 2Cr(OH)_3_↓, (3)

Fe_2_(SO_4_)_3_ + 3Ca(OH)_2_ = 3CaSO_4_↓ + 2Fe(OH)_3_↓. (4)

During the precipitation of iron (III) hydroxide, the most complete transition of water impurities into the solid phase occurs due to the co-precipitation and adsorption on the surface of freshly precipitated iron hydroxide. The presence of CaSO_4_ crystals in the pulp contributes to the improvement of the sedimentation and filtration processes. The use of alkali solutions for the precipitation of trivalent chromium in the form of hydroxide is also possible:

Cr_2_(SO_4_)_3_ + 6NaOH = 3Na_2_SO_4_ + 2Cr(OH)_3_↓. (5)

Such methods of processing of waste chromium-containing solutions result in sulfates remaining in the system after the separation of the sediments. Sulfates are secondary waste products which require disposal.

We propose to use electrodialysis with bipolar membranes for the conversion of these solutions. The acid and alkali obtained during the conversion can be used during earlier stages of the chemical treatment of wastewater containing Cr_2_O_7_^2−^ (to adjust the pH at the stage of Cr_2_O_7_^2-^ reduction, for the precipitation of Cr(OH)_3_).

Bipolar ion-exchange membranes consist of a cation-exchange layer (CEL) tightly coupled with an anion-exchange layer (AEL). At the boundary of these layers, when an electric potential gradient is applied to the system, the irreversible dissociation of water molecules occurs, and the dissociation products leave the reaction zone. Some studies [15,16,17,18] demonstrated that the ionogenic groups of the membranes participate in water dissociation and are able to influence its rate. The use of metal salts as catalytic additives in the process of water dissociation was proposed by Simons [19]. The authors of [20,21] supplemented the known series of ionogenic groups with catalytic influence on water dissociation with some transition metal hydroxides and proposed a mechanism of water dissociation with the participation of metal hydroxides:

Me(OH)_n_ ⇔ Me(OH)^+^_n−1_ + OH^−^, (6)

Me(OH)^+^_n−1_ + 2H_2_O ⇔ Me(OH)_n_ + H_3_O^+^. (7)

Both inorganic substances, including metals introduced into the ion exchanger in various forms (elementary substance, oxides, hydroxide) [22,23,24,25,26], and organic compounds [27] can act as catalysts.

The MB-2 bipolar membrane is characterized by a very high operating voltage of water dissociation and a low current efficiency of acids and bases [28]. It is known that Cr(OH)_3_ has the highest catalytic activity in the dissociation of water in the bipolar region [20,21]. The treatment of sodium sulfate solutions—the secondary effluent of the processing of chromium-containing wastewater—can be a good application for such membranes. In this regard, it is of interest to study the electrochemical behavior of chromium-modified bipolar membranes during the conversion of sodium sulfate.

The goals and objectives of this study were the modification of a commercially available bipolar membrane with chromium (III) hydroxide; an investigation of the features of the distribution of the modifier over the volume of the sample; an evaluation of the influence of the modifier on the characteristics of the salt (sodium sulfate) conversion process for the possible suggestion of a variant of the practical application of electrodialysis based on a chromium-modified bipolar membrane.

## 2. Materials and Methods

The MB-2 membrane was modified chemically [29]. This procedure included the following steps: treatment of the membrane with alkali (sodium hydroxide solution); washing the membrane in water; treatment of the membrane with a solution of chromium (III) sulfate (0.5 mol-equiv/dm^3^); washing the membrane with water; treatment of the membrane with a sodium hydroxide solution; washing the membrane with water again.

An analysis of the surface morphology and volume of the modified membrane was performed by means of electron microscopy using a JSM-6380LV (JEOL, Tokyo, Japan) scanning electron microscope and a scanning electron microscope with a Quanta Nova field-emission cathode (FEI, Eindhoven, The Netherlands) in the mode of secondary electrons. The chemical composition of the membrane material was assessed by an energy-dispersive X-ray analyzer (EDAX, Mahwah, USA).

The effect of the modifier, chromium (III) oxide, on the mechanism of current flow through the MB-2 cation-exchange layer was evaluated using an extended three-wire model for the description of the parameters of ion-exchange materials [30]. For this, the electrical conductivity of the MK-40 membrane, which is a cation-exchange layer of the MB-2 bipolar sample, was considered. In this membrane, as in MB-2, the modifier was introduced by the chemical method, by the sequential treatment of the ion exchanger in the sodium form with a salt solution of chromium (III) and sodium hydroxide (a detailed description is given above). The electrochemical impedance of MK-40 samples was measured using the contact-difference method [31] at an alternating current frequency of 100 kHz (Tesla BM 507 impedance meter). The active component of the membrane impedance was determined, and the specific electrical conductivity of the membrane, k_m_, was calculated according to the formula:

(8) $$k_{m}=\frac{d}{RA},$$

where d is the thickness of the membrane (determined using a micrometer), cm; R is the electrical resistance of the membrane, Ohm; A is the area of the membrane or electrode, cm^2^.

**Figure 2:**
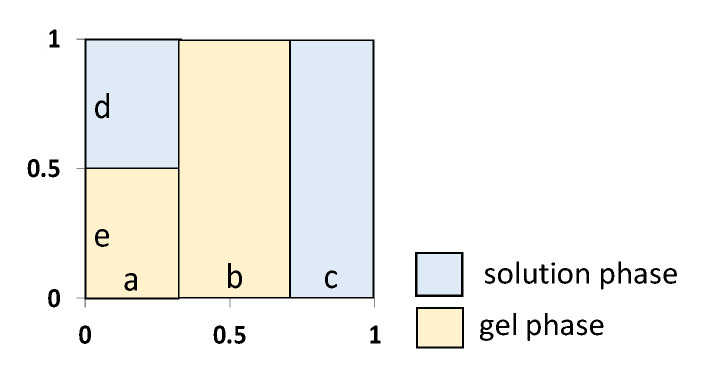
Current transfer paths in an ion-exchange material in an extended three-wire model [30].

The extended three-wire model makes it possible to find structural (f_1_, f_2_, α) and geometric (a, b, c, d, e) sample parameters based on the concentration dependence of the electrical conductivity of an ion-exchange material [30]. The parameter b corresponds to the fraction of the current passing through the gel phase of the membrane, the parameter c corresponds to the intergel solution, the parameter a corresponds to the mixed gel –solution channel, the parameters d and e are fractions of solution and gel, respectively, in the mixed channel a. The parameters f_1_ and f_2_ are the volume fractions of the gel and intergel phases; α is a parameter that reflects the mutual arrangement of phases in the material with respect to the direction of the current and takes values from +1 (parallel arrangement) to −1 (series). The value α should be taken into account for the description of the diffusion permeability of ion-exchange membranes. This method of parametrization of ion-exchange membranes is based on an approach that was developed for ion-exchange columns and resins. The current flows through the ion-exchange material through three parallel channels (Figure 2): sequentially through ionite and solution, only through ionite, and only through solution.

In addition, this approach is based on the use of a microheterogenic model and the theory of generalized conductivity of structurally inhomogeneous media. According to the microheterogenic model, two pseudophases are distinguished in the ion-exchange membrane, i.e., a gel, in which the current is carried only by counterions, and an inter-gel, in which the current is carried by both cations and anions. The gel phase is combined and contains all the components of the ion-exchange material with the exception of the equilibrium solution. The inter-gel phase consists of gaps filled with a solution whose properties are identical to those of an equilibrium solution.

**Figure 3:**
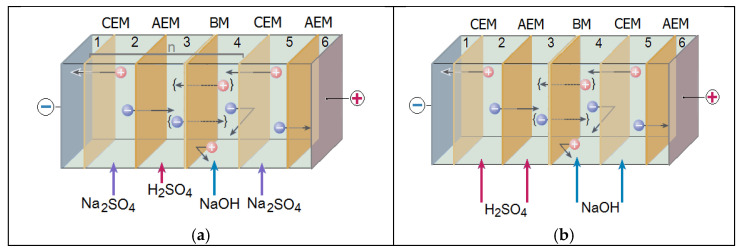
Diagram of an electrodialysis apparatus with a three-chamber unit cell for obtaining sulfuric acid and sodium hydroxide solutions: (**a**)—for the first and second variants of the experiment; (**b**)—for the third variant of the experiment (description is in the main text). CEM, cati on-exchange membrane, AEM, anion-exchange membrane, BM, bipolar membrane; n is an elementary repeating fragment.

The experiment on the conversion of sodium sulfate was carried out in galvanostatic mode in the electrodialysis apparatus with a three-chamber unit cell. This variant of the experiment involved the alternation of three types of membranes: cation-exchange, anion-exchange, and bipolar membranes (Figure 3). 

In the electrodialyzer, the MB-2 membrane modified with chromium (III) hydroxide (designated as MB-2-Cr) and the commercially available MB-3 and MB-2 bipolar membranes were used [32]. MK-40 and MA-41 were used as monopolar membranes [32]. The characteristics of these membranes are shown in Table 1.

The working area of one membrane was 14 cm^2^, the intermembrane distance in the channels was 1 mm. The experiment was carried out in three variants. 

First variant. In the electrodialyzer (Figure 3, a, chambers 2, 5) in a single-pass mode (without recycle), a solution of sodium sulfate with a concentration of 0.5 mol/dm^3^, sulfuric acid (0.05 mol/dm^3^), and a sodium hydroxide solution (0.1 mol/dm^3^) were delivered to chambers 3 and 4. The rate of the saline flow was 20 cm^3^/min, and that of the acid and base solutions was –1 cm^3^/min. The number of elementary cells was n = 1. Such a saline flow rate in the desalination chambers allowed the process to be carried out in under-limit current modes. The formation of hydrogen and hydroxide ions occurred under the influence of the direct current in the bipolar membrane, and these ions migrated into the acidic (3) and basic (4) chambers. From the salt desalination sections (2 and 5), sulfate ions and sodium ions migrated through the anion- and cation-exchange membranes into the acidic and alkaline chambers, respectively. The process was carried out in galvanostatic mode: the current density was set (14.3–64.3 mA/cm^2^), and after a steady state was established in the system, the samples were analyzed. 

The second variant of the experiment differed from the first one in that the process was carried out using a circulating batch system (with recycle): certain fixed volumes of salt, acid, and base solutions repeatedly passed through the electrodialyzer and returned to the storage tanks. The flows supplied to the chambers are shown in (Figure 3, a). The flows through the stacks were parallel. The volume averaged electrolyte velocity in each chamber was 20 cm^3^/min. This version of the experiment allowed us to control the saline flow rate in the desalination chambers and ensure conditions under which the current did not exceed the limit value. The volume of circulating saline solution was 1 dm^3^, the volumes of the acid and base solutions were 0.1 dm^3^ each. The number of elementary cells was n = 3. The process was also carried out in the galvanostatic mode at 64.3 mA/cm^2^.

The third variant of the experiment was associated with the need to control the degree of contamination of the acid and base solutions with salt ions. The point is that along with the target ion flows, there are also undesirable SO_4_^2−^ ion flows through the bipolar membrane from chamber 3 to the main chamber 4 and Na^+^ ion flows from chamber 4 to chamber 3 (shown in Figure 3 by the dotted arrow and curly brackets {}). These were crossover flows of salt ions [33]. These flows reduced the current efficiency of the acids and bases and contaminated the target products. For the determination of sodium cations in chamber 3 and sulfate anions in chamber 4 that crossed through the bipolar membrane, the experiment was carried out according to the scheme shown in Figure 3b. In contrast to the first and second variants, acid and alkali solutions, respectively, were supplied to chambers 2 and 5, as well as to 3 and 4. This variant allowed avoiding the influence of the non-ideal selectivity of monopolar membranes in the studied process. Other characteristics of the experiment (current mode, feed rates, single-pass process) were identical to those of the first variant.

The concentration of hydrogen and hydroxide ions in the acidic and basic chambers was determined using acid–base titration [34]; the concentration of sodium (chamber 3) and sulfate (chamber 4) ions was determined by direct potentiometry using ion-selective electrodes [35,36]. Such characteristics of the process as current efficiency (*η*, dimensionless value), flux density of hydrogen and hydroxyl ions, formed inside the bipolar membrane (*J*, mol/(m^2^·s)), specific energy consumption (*W*, W·h/kg) (experiment according to variants 1 and 2) were calculated based on the results of experiments:

(9) $$\eta =\frac{F}{n\cdot i\cdot A}\Delta c\cdot \omega ,$$

(10) $$J=\frac{\Delta c\cdot \omega }{n\cdot A},$$

(11) $$W=\frac{I\cdot U\cdot \tau }{m}.$$

Here, Δ*c* is the change in the concentration of hydrogen H^+^ and hydroxyl OH^−^ ions at the inlet and outlet of the concentration chamber, mol/m^3^; *i* is current density, A/m^2^; *I* is the operating value of the current strength, A; *U* is the operating voltage, V; *τ* is the time of the experiment, h; *m* is the mass of the target product (acid or alkali) obtained during the experiment, kg; *ω* is the volumetric flow rate of the solution, m^3^/s; *n* is the number of elementary cells (chambers of type 2, 3, 4) in the electrodialyzer; *A* is the active membrane area, m^2^; *F* is the Faraday constant, 96485 C/mol. The degree of acid and base contamination with salt ions that crossed the membrane (*r_cr_*, %) was determined using the formula (12):

(12) $$r_{\mathrm{cr}\;}({\mathrm{Na}}^{+})=\frac{\nu ({\mathrm{Na}}^{+})}{\nu (H^{+})}\cdot 100;{\;r}_{\mathrm{cr}\;}\left({\mathrm{SO}}_{4}^{2-}\right)=\frac{\nu ({\mathrm{SO}}_{4}^{2-})}{\nu ({\mathrm{OH}}^{-})}\cdot 100,$$

where ν(_Na+_) is the amount of Na^+^ cations (in moles) that crossed into the acidic chamber for a certain time, ν(_H+_) is the amount of H^+^ ions (in moles) generated during the same time, ν(SO_4_^2−^) is the amount of SO_4_^2−^ anions (in moles) that crossed into the alkali chamber for a certain time, and ν(_OH−_) is the amount of OH^−^ ions (in moles) generated during the same time [33].

## 3. Results and Discussion

When the MB-2 and MK-40 membranes were treated with a chromium salt solution, metal ions entered the cation exchanger and were localized near ionogenic centers as counterions [29]. An ion-exchange saturation reaction occurred:

3R-SO_3_^−^Na^+^ + 1/2Cr_2_(SO_4_)_3_ = (R-SO_3_^−^)_3_Cr^3+^ + 3/2Na_2_SO_4_. (13)

After treatment of the membrane with a sodium hydroxide solution, a sparingly soluble Cr(OH)_3_ was formed near the ionogenic groups in the polymer:

(R-SO_3_^−^)_3_Cr^3+^ + 3NaOH = [R-SO_3_^−^Na^+^]_3_·Cr(OH)_3_. (14)

In this case, the cation exchanger (the cation-exchange layer of a bipolar membrane or monopolar cation-exchange membrane) acquired the gray-green color characteristic of chromium (III) hydroxide. 

Such treatment resulted in the following reactions in the MB-2 anion-exchange layer:

6(R-CH_2_-N^+^(CH_3_)_3_)_2_OH^−^ + Cr_2_(SO_4_)_3_ = 3(R-CH_2_-N^+^(CH_3_)_3_)_2_SO_4_^2−^·2Cr(OH)_3_, (15)

3(R-CH_2_-N^+^(CH_3_)_3_)_2_SO_4_^2−^·2Cr(OH)_3_ + 6NaOH = 3[(R-CH_2_-N^+^(CH_3_)_3_)_2_OH^−^]_2_·2Cr(OH)_3_ + 3Na_2_SO_4_. (16)

It was shown in [37] that, under these modified conditions, Cr(OH)_3_ is present in the membrane in a nanoscale form, with a gradient distribution over the ionite particles [38]: the concentration of Cr(OH)_3_ decreased exponentially from the surface deep into the ionite granules.

**Figure 4:**
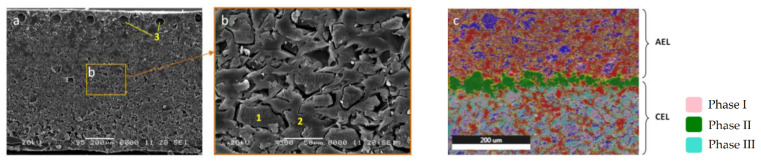
Cross section of the bipolar membrane MB-2-Cr: electron micrograph (**a**,**b**) and phase analysis (**c**). 1, Ionite particles; 2, polyethylene; 3, reinforcing fiber; AEL, anion-exchange layer; CEL, cation-exchange layer; I, II, III are phases with different composition.

Micrographs of the cross section of MB-2-Cr are shown in Figure 4. Ionite particles (1, Figure 4) distributed in an inert plasticizer—polyethylene (2, Figure 4)—and fragments of a reinforcing fiber (3, Figure 4) can be identified. The elemental composition of phases containing chromium for MB-2-Cr is shown in Table 2. The total content of chromium in the considered section of the membrane was 0.51 at. %, and most chromium was concentrated in Phase II.

**Figure 5:**
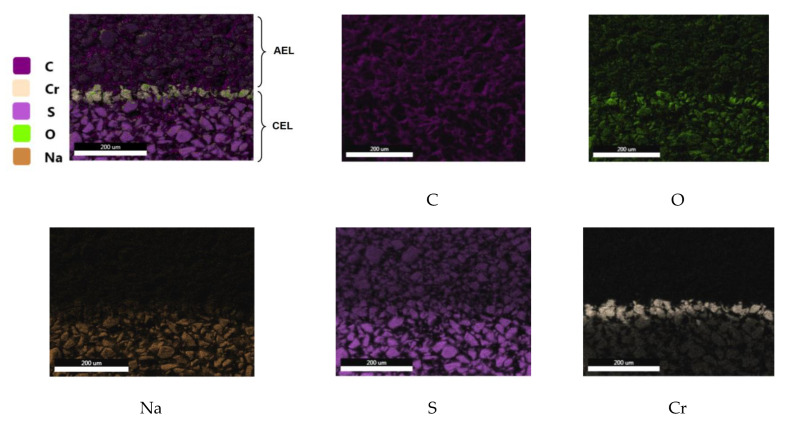
Elemental analysis of a cross section of a bipolar MB-2-Cr membrane, AEL, anion-exchange layer, CAL, cation-exchange layer.

The distribution of elements in the considered MB-2-Cr section is shown in Figure 5. It can be seen that the boundary of the cation- and anion-exchange layers of the membrane were mainly enriched in chromium and oxygen and depleted in sodium and sulfur, in comparison with the volume of the cation-exchange layer. Chromium (III) hydroxide was distributed in a layer of 30–50 μm, at the boundary of the cation and anion exchanger.

**Figure 6:**
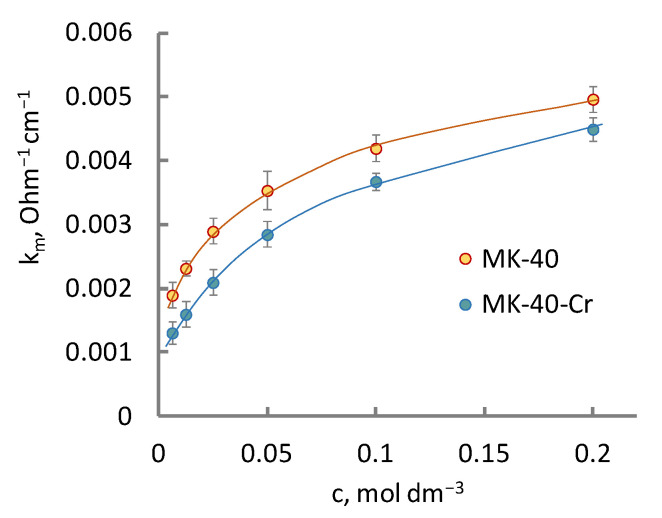
Concentration dependence of the electrical conductivity of MK-40 in Na_2_SO_4_ solutions.

**Figure 7:**
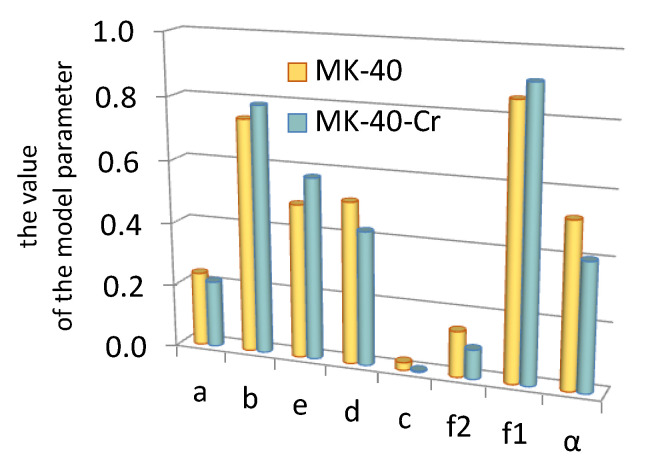
Model parameters characterizing the paths of current transfer (a, b, c, d, e), volume fraction of the gel (f_1_) and intergel (f_2_) phases of the MK-40 membrane and orientation of the phases in the direction of the current flow (α). 1, unmodified MK-40 sample; 2, MK-40-Cr membrane containing chromium oxide (III).

For the assessment of the effect of the modifier on the cation-exchange layer of the bipolar membrane, the electrical conductivity of only the MB-2 cation-exchange layer of the MK-40 membrane was studied before and after its modification with chromium (III) hydroxide. The obtained concentration dependences of the electrical conductivity of the MK-40 and MK-40-Cr membranes are shown in Figure 6. After the modification, the electrical conductivity of the membrane decreased. The parameters a, b, c, d, e, f_1_, f_2_, and α determined from the concentration dependence of the specific electrical conductivity of the membrane are shown in Figure 7 (extended three-wire model [30]). 

The analysis of the model parameters showed that when a modifying additive was introduced into MK-40, the fraction of the current passing through the channel c and the intergel phase (f_2_) decreased. The decrease in c and f_2_ was due to the substitution of part of the free solution in the pore volume by the modifier. The decrease in the α parameter indicated the disordering effect of the modifier on the relative position of the conductive phases of the membrane. The symbatic change of c and f_2_ was noted in [39], where the change in the structural parameters of membranes after current–temperature exposure was studied. The influence of the modifier on the proportion of the intergel phase participating in the current transfer in the ion-exchange membrane was revealed in a previous study [40]. Here, the authors worked with a two-phase model of a heterogeneous membrane [41] and, based on this model, proposed a microstructural description of modified ion-exchange membranes.

**Figure 8:**
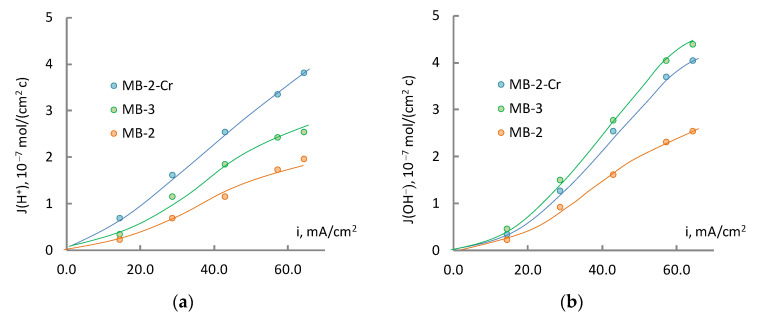
Flux density of hydrogen (**a**) and hydroxyl (**b**) ions as a function of current density during electrodialysis with bipolar membranes.

The analysis of the current dependences of the densities of hydrogen and hydroxyl ion fluxes obtained by the electrochemical conversion of sodium sulfate (Figure 8) using different types of bipolar membranes showed that the use of modified MB-2-Cr samples allowed obtaining target products with concentrations close to the concentrations of these substances obtained using MB-3. The MB-3 bipolar membrane contains phosphonic acid groups in the cation-exchange layer, which possess the highest catalytic activity with respect to water dissociation [18]. The modification of the MB-2 membrane with chromium (III) hydroxide also allowed reducing the specific energy consumption in the production of acids and bases (Table 3).

When working in the recycling mode, it was possible to obtain a sufficiently high concentration of alkali (Table 3). However, the acid concentration was not as high as could be expected. This was probably due to the more active transfer of protons into the saline solution stream. This is one of the problems of standard anion-exchange membranes. To solve this problem, research is being conducted on the development of anion-exchange membranes with proton blocking properties [42].

**Figure 9:**
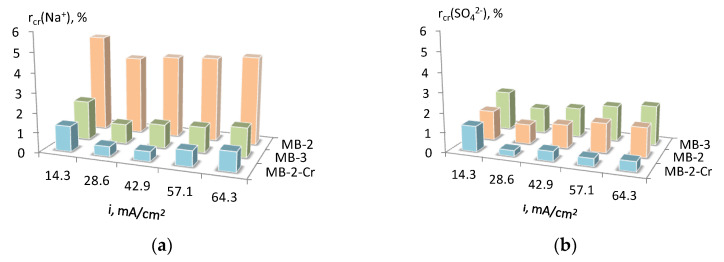
Degree of acid (**a**) and base (**b**) contamination with salt ions that crossed the membrane (determined using formula (12)).

The degree of contamination of the target products with salt ions is shown in Figure 9. The modification of the MB-2 membrane contributed to the reduction of the transfer of salt ions through MB-2-Cr and resulted in more pure target products. The low selectivity of the MB-3 membrane with respect to sulfate ions, associated with the presence of weakly acidic phosphoric acid groups in its cation-exchange layer, should be noted. In this context, membranes with strongly acidic functional groups are advantageous. This is probably the reason for the lower H_2_SO_4_ concentration obtained for MB-3 in comparison with MB-2-Cr. (Figure 8a).

Thus, the volumetric modification of the cation-exchange layer of the MB-2 bipolar membrane with chromium (III) hydroxide allowed reducing the contamination of the target products with salt ions and obtaining higher concentrations of acid and base with lower power consumption.

Based on the data obtained, we can recommend the use of chromium-modified bipolar membranes for the electrodialysis treatment of secondary waste solutions formed during the processing of chromium-containing wastewater. In this case, the possible degradation of the modifier is not critical. In addition, the modification of industrial bipolar membranes with chromium can be carried out directly in industries where there is chromium-containing wastewater; obviously, this approach is more cost-effective.

**Figure 10:**
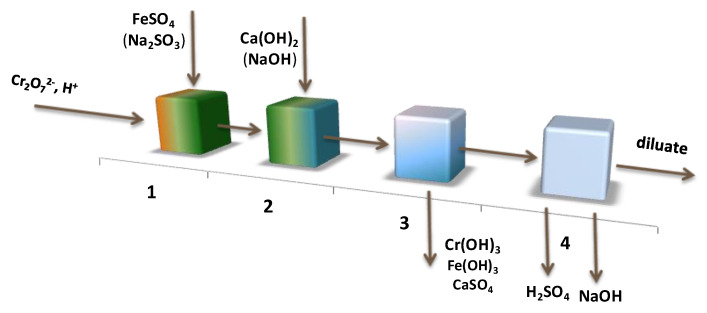
Scheme of the treatment of chromium-containing wastewater with the introduction of the electromembrane processing stage for the conversion of a salt solution. Designations are given in the text.

The scheme of the improved process for the treatment of chromium-containing wastewater is shown in Figure 10.

Wastewater containing Cr_2_O_7_^2-^ enters the chromium recovery stage (block 1). Next, is the neutralization stage (block 2), then the precipitation of sparingly soluble hydroxides (block 3) and the processing of the salt solution in an electrodialysis synthesizer with bipolar membranes (block 4). The resulting diluate and acid and alkali solutions can be used during the earlier stages of the chromium wastewater treatment process. 

The treatment of sodium sulfate by electrodialysis with bipolar chromium-modified membranes proposed in this study can also be useful to improve the technology considered in [43], where the chromium removal process involves the reduction of Cr(VI) to Cr(III) at the cathode and then the adsorption of Cr(III) at the cathode. Sulfates as secondary stock products are also formed during the reduction of pyrite with bichromate [44].

The use of a chromium-modified bipolar membrane can be recommended in a number of applications, for example, as a continuation of a previous study [45], where the isolation of Cr(III) from an aqueous solution in the form of Na_2_CrO_4_ after the oxidation of Cr(III) to Cr(VI) with H_2_O_2_ in an alkaline medium in an electrodialyzer with bipolar membranes was proposed, or in relation to another study [46], where the extraction of Cr(VI) from chromite ore processing residual was proposed.

## 4. Conclusions

A chromium-modified bipolar ion-exchange membrane was obtained by the sequential chemical treatment of an industrial membrane with a chromium (III) salt and alkali. The structure of the membrane and its influence on the characteristics of the electrodialysis conversion of sodium sulfate were investigated.

Chromium (III) hydroxide was found to be distributed in the layer of 30–50 μm, at the boundary of the cation and anion exchanger. When the modifier was introduced into the bipolar region, the acid and alkali performance during the conversion of sodium sulfate increased, and the specific energy consumption decreased almost twice as compared to the unmodified sample.

It was shown that the chemical modification of the MB-2 bipolar membrane with chromium (III) oxide allowed not only an increase in the yield of acids and bases and a reduction in the energy consumption of the process, but also a reduction in the degree of contamination of the target products with salt ions by three to four times. The latter was associated with the limitation of the diffusion transfer of the salt, limited by the transfer of coions, and was a consequence of a change in the current transfer paths in the modified sample.

When implementing the process in the recycle mode, with the conversion of sodium sulfate at an initial concentration of 0.5 mol/dm^3^, solutions of sulfuric acid and sodium hydroxide with concentrations of 0.47 and 1.5 mol/dm^3^, respectively, were obtained. This was achieved with a volume ratio of salt to acid (alkali) solutions equal to 10:1.

The use of a chromium-modified bipolar membrane in the electrodialysis treatment of both chromium-containing wastewater and secondary stock solutions obtained during the processing of chromium-containing wastewater is recommended.

## Figures and Tables

**Table 1:** Characteristics of the ion-exchange membranes used in the study [32].

Heterogeneous Membranes		Functional Groups	Thickness, mm
monopolar	MK-40	-SO_3_H	<0.45
	MA-41	-N^+^(CH_3_)_3_	<0.45
bipolar	MB-2	-SO_3_H -N^+^(CH_3_)_3_	0.5–0.8
	MB-3	-PO_3_H_2_ -N^+^(CH_3_)_3_	0.5–0.8

**Table 2:** Elemental composition of the phases.

Phase	Atomic Fraction of an Element in Each Phase, %				
	C	O	S	Cr	Na
Phase I	68.3	9.4	11.5	1.4	9.4
Phase II	70.9	12.8	5.7	6.0	4.6
Phase III	67.6	15.1	5.9	0.8	10.6

**Table 3:** Results of experiments on the conversion of sodium sulfate (0.5 mol/dm^3^) at 64 mA/cm^2^.

Process	Without Recycling						With Recycling	
**Membranes**	MB-2		MB-3		MB-2-Cr		MB-2-Cr	
**Products**	NaOH	H_2_SO_4_	NaOH	H_2_SO_4_	NaOH	H_2_SO_4_	NaOH	H_2_SO_4_
Δ*c*, mol-equiv /dm^3^	0.22	0.17	0.38	0.22	0.35	0.33	1.50	0.95
η, %	38	30	66	38	61	57	51	31
W, kW⋅h/kg	34.3	38.6	15.9	23.8	19.3	17.9	18.1	23.2

## Data Availability

Not applicable.
